# Preoperative serum creatinine changes and acute kidney injury in patients underwent cardiac surgery

**DOI:** 10.3389/fmed.2025.1584418

**Published:** 2026-01-08

**Authors:** Bo Jiang, Yi Hao, Meiping Wang, Liyan Chen, Zhenhua Zhang, Lin Chen, Ning He, Yueling Chen, Shuai Zhu, Li Jiang, Haiping Yang

**Affiliations:** 1 Intensive Critical Unit, Beijing Luhe Hospital, Capital Medical University, Beijing, China; 2 Department of Cardiac Surgery, Beijing Luhe Hospital, Capital Medical University, Beijing, China; 3 Intensive Critical Unit, Xuanwu Hospital, Capital Medical University, Beijing, China; 4 Beijing Water Conservancy and Hydropower School, Beijing, China

**Keywords:** acute kidney injury, cardiac surgery, preoperative assessment, mortality, preoperative serum creatinine

## Abstract

**Introduction:**

Preoperative serum creatinine fluctuations are common in open-heart surgery, and their association with postoperative acute kidney injury (AKI) and the combined impact on patient outcomes warrant further investigation.

**Methods:**

This retrospective cohort study assessed patients undergoing open-heart surgery. Preoperative serum creatinine changes (ΔScr) were calculated as the difference between the serum creatinine value within 48 h before surgery and baseline. Patients were categorized into three groups based on ΔScr: negative (< 0 mg/dl), normal (0–0.3 mg/dl), and elevated (≥0.3 mg/dl). Multivariable logistic regression and restricted cubic spline models were used to analyze the clinical outcomes.

**Results:**

Of the 560 patients included, 40.2% developed AKI. There were significant increases in the odds of AKI [adjusted odds ratio (AOR), 1.51; 95% CI, 1.32–1.72, per 0.1 mg/dl increase], severe AKI (AOR, 1.45; 95% CI, 1.24–1.70), and AKI non-recovery (AOR, 1.37; 95% CI, 1.19–1.59). In AKI patients, negative ΔScr was associated with a higher rate of in-hospital mortality and ICU LOS >72 h compared to without AKI, while elevated ΔScr showed no significant differences. In addition, negative ΔScr was associated with a higher risk of in-hospital mortality (AOR, 4.50; 95% CI, 1.00–20.15) and ICU LOS >72 hours (AOR, 2.81; 95% CI, 1.13–6.96) compared with normal ΔScr. No significant associations were observed with elevated ΔScr. In contrast, among patients without AKI, neither negative nor elevated ΔScr were associated with in-hospital mortality or prolonged ICU LOS.

**Conclusions:**

In this retrospective study of elective cardiac surgery, negative changes in preoperative serum creatinine were less likely to development of AKI. However, patients with negative changes who developed postoperative AKI had a higher risk of in-hospital mortality and prolonged ICU stays. No significant associations with these outcomes were observed with elevated changes.

## Introduction

Acute kidney injury (AKI) is a common complication in patients undergoing open-heart surgery. It significantly contributes to increased morbidity and mortality and is associated with a longer intensive care unit (ICU) length of stay (LOS) and extended hospitalizations (1–3). Even patients who fully recover kidney function experience a heightened risk of chronic kidney disease (CKD) and mortality in subsequent years (4, 5). Despite extensive research efforts over the past few decades, progress in treating established AKI remains limited (6, 7). Prevention of AKI is crucial for improving patient outcomes.

Preoperative renal dysfunction is a prevalent concern in patients undergoing cardiac surgery, with approximately 75% of patients experiencing elevated preoperative serum creatinine (8). Numerous prior studies have established that increased preoperative serum creatinine is a powerful predictor of cardiac surgery associated AKI (9–11). Recent findings suggest that dynamic changes in serum creatinine may offer more valuable insights than a single measurement. However, the impact of preoperative creatinine changes on AKI risk has been less thoroughly studied. In addition, preoperative serum creatinine levels are associated with postoperative adverse outcomes (12–17). A study demonstrated that acute creatinine elevations from baseline are independently associated with increased rates of mortality, and prolong ICU LOS (18). Although the association between AKI and poor outcomes in cardiac surgery patients is well established, the impact of preoperative serum creatinine changes on postoperative adverse outcomes in patients who develop AKI remains unclear.

The primary objective of this study is to evaluate the relationship between preoperative serum creatinine changes and postoperative AKI risk in patients undergoing open-heart surgery. The secondary objective is to examine how these changes correlate with clinical outcomes, including in-hospital mortality and ICU LOS, in patients who develop AKI following cardiac surgery.

## Methods

### Study population

This retrospective observational cohort study was conducted at Beijing Luhe Hospital, Capital Medical University, from January 2015 to December 2021. Eligible patients met the following criteria: (1) aged ≥18 years, (2) underwent open-heart surgery, and (3) were admitted to ICU following surgery. Patients were excluded if they met any of the following criteria: (1) underwent emergency surgery, (2) had a baseline estimated glomerular filtration rate (eGFR) of < 60 ml/min/1.73 m^2^ (19), (3) had only one preoperative serum creatinine measurement with no preadmission creatinine available, or (4) died or were discharged less than 24 h after the operation. The study protocol and waiver of informed consent were approved by the Medical Ethics Committee of Beijing Luhe Hospital, Capital Medical University (approval reference number: 2022-KY-070).

### Data collection

Data collected included patient characteristics, comorbidities, and preoperative medications. Surgical parameters, ICU LOS, and hospital mortality were also documented. Additionally, the European System for Cardiac Operative Risk Evaluation II (EuroSCORE II), a predictive model for operative mortality in cardiac surgery, was calculated (20). Cardiac function was assessed using the New York Heart Association (NYHA) classification (21). Contrast agent use for percutaneous coronary interventions was recorded within one month preoperatively.

### Exposure

The exposure in this study was the change in preoperative serum creatinine (ΔScr), calculated as the difference between the serum creatinine value within 48 h before surgery and baseline. Baseline serum creatinine was defined as the lowest serum creatinine value recorded within the 6 months preceding hospital admission or, if unavailable, the serum creatinine level at admission. Patients were categorized into three groups based on ΔScr: negative ΔScr (ΔScr < 0 mg/dl), normal ΔScr (ΔScr 0–0.3 mg/dl), elevated ΔScr (ΔScr ≥0.3 mg/dl). These cut-points were based on the Kidney Disease Improving Global Outcomes (KDIGO) definition of AKI, in which stage 1 is characterized by an increase of 0.3 mg/dl or greater (22).

### Outcome measures

The primary outcome was AKI, severe AKI and AKI non-recovery after surgery. AKI was defined according to KDIGO criteria: stage 1, an absolute increase in serum creatinine of ≥0.3 mg/dl or a 1.5– to 2-fold increase from baseline, or urine output < 0.5 ml/kg/h for 6–12 h; stage 2, a 2– to 3-fold increase from baseline, or urine output < 0.5 ml/kg/h for ≥12 h; and stage 3, a serum creatinine ≥4.0 mg/dl, a greater than 3-fold increase from baseline, or the requirement for kidney replacement therapy, or urine output < 0.3 ml/kg/h for ≥24 h, or anuria for ≥12 h (22). Severe AKI was defined as KDIGO stages 2 or 3. AKI non-recovery was defined as failure of the serum creatinine level to decrease to less than 150% of the baseline from AKI onset within 7 days (23). Secondary outcomes were ICU LOS >72 h and in-hospital mortality.

### Statistical analysis

Continuous data were presented as median and interquartile range (IQR), while categorical data were reported as counts with percentages. The nonparametric Wilcoxon rank-sum test was used to compare continuous variables, and the Kruskal–Wallis test was employed to compare more than two groups because none of the variables satisfied the normality assumptions for parametric tests. Categorical variables were compared using the Chi-squared test. Multivariable logistic regression models were employed to analyze the primary and secondary outcomes with ΔScr as a continuous variable. The primary and secondary outcomes were assessed using logistic regression analysis, adjusted for key demographic and clinical factors associated with ΔScr. Restricted cubic spline curves were used to assess the nonlinear associations between ΔScr and AKI development, as well as in-hospital mortality and ICU LOS >72 h. To test robustness and minimize confounding from preoperative kidney impairment, we performed a sensitivity analysis excluding patients with serum creatinine ≥1.5 times baseline within 48 hours before surgery. Associations between ΔScr categories and outcomes were reassessed. Statistical analyses were performed using R software (ver. 4.1.2; R Foundation for Statistical Computing, Vienna, Austria). A two-tailed P-value of less than 0.05 was considered statistically significant for all analyses.

## Results

### Study participants

Among the 634 patients undergoing open-heart surgery, 560 were included in the final analysis. Patients were categorized into three groups according to ΔScr as follows: 127 (22.7%), negative ΔScr; 358 (63.9%), normal ΔScr; and 75 (13.4%), elevated ΔScr (Supplementary Figure S1). The baseline characteristics of the study population across the ΔScr categories are shown in Table 1. Overall, the median age was 64 years (IQR, 57–68 years), and 63.4% of the patients were male. The median ΔScr was 0.1 mg/dl (IQR, 0.0–0.2 mg/dl), as shown in Supplementary Figure S2. In the cohort, 30.5% of the patients were classified as NYHA class III/IV, and the median EuroSCORE II was 2.0 (IQR, 1.0–3.0), with higher scores indicating greater mortality risk.

**Table 1:** Characteristics of patients.

**Characteristic**	**All subjects (*n*=560)**	**Normal ΔScr (*n* = 358)**	**Negative ΔScr (*n* = 127)**	**Elevated ΔScr (*n* = 75)**	***P*-value**
Age, median (IQR), year	64 (57, 68)	64 (57, 69)	63 (56, 67)	64 (60, 69)	0.276
Male, *n* (%)	355 (63.4)	228 (63.7)	80 (63)	47 (62.7)	0.981
EuroSCORE II, median (IQR)	2.0 (1.0, 3.0)	2.0 (1.0, 3.0)	2.0 (1.0, 3.0)	3.0 (2.0, 4.0)	0.004
Hypertension, *n* (%)	333 (59.5)	209 (58.4)	72 (56.7)	52 (69.3)	0.164
Diabetes mellitus, *n* (%)	163 (29.1)	97 (27.1)	44 (34.6)	22 (29.3)	0.274
Stroke, *n* (%)	76 (13.6)	76 (13.6)	22 (17.3)	41 (11.5)	0.15
NYHA class III/IV, *n* (%)	171 (30.5)	100 (27.9)	36 (28.3)	35 (46.7)	0.005
ACEIs or ARBs, *n* (%)	265 (47.3)	174 (48.6)	62 (48.8)	29 (38.7)	0.272
Diuretics, *n* (%)	331 (59.1)	224 (62.6)	64 (50.4)	43 (57.3)	0.053
Statins, *n* (%)	282 (50.4)	174 (48.6)	71 (55.9)	37 (49.3)	0.361
NSAIDs, *n* (%)	274 (48.9)	171 (47.8)	67 (52.8)	36 (48)	0.617
Contrast agent, *n* (%)^a^	474 (84.6)	304 (84.9)	106 (83.5)	64 (85.3)	0.912
Baseline eGFR, median (IQR), ml/min/1.73 m^2^	89.8 (80.0, 97.7)	91.6 (83.3, 99.0)	87.6 (79.6, 96.2)	82.0 (72.8, 93.8)	< 0.001
Baseline serum creatinine, mg/dl	0.83 (0.72, 0.92)	0.80 (0.70, 0.89)	0.85 (0.76, 0.95)	0.87 (0.78, 0.98)	< 0.001
ΔScr, mg/dl^b^	0.1 (0.0, 0.2)	−0.1 (−0.1, 0.0)	0.1 (0.1, 0.2)	0.4 (0.4, 0.5)	< 0.001
Preoperative length of stay, median (IQR), day	14 (9, 21)	14 (9, 20)	14 (9, 20)	19 (13, 30)	< 0.001
**Type of surgery**, ***n*** **(%)**					
Coronary artery bypass graft surgery	278 (49.6)	185 (51.7)	69 (54.3)	24 (32)	0.003
Valve surgery	212 (37.9)	128 (35.8)	43 (33.9)	41 (54.7)	
Combined	48 (8.6)	31 (8.7)	7 (5.5)	10 (13.3)	
Other	22 (3.9)	14 (3.9)	8 (6.3)	0 (0)	
Surgery time, median (IQR), min	314 (260, 365)	310 (260, 360)	305 (257, 353)	340 (283, 405)	0.004
Cardiopulmonary bypass, *n* (%)	300 (53.6)	188 (52.5)	58 (45.7)	54 (72)	0.001
Intraoperative blood product transfusion, median (IQR), ml	1,105 (252, 1,686)	1,100 (260, 1,695)	720 (169, 1,490)	1,434 (1,035, 1,851)	< 0.001

EuroSCORE II, European System for Cardiac Operative Risk Evaluation II; ACEI, angiotensin-converting enzyme inhibitor; ARB, angiotensin receptor blocker; NYHA, New York Heart Association; eGFR, estimated glomerular filtration rate; NSAID, non-steroidal anti-inflammatory drugs; IQR, interquartile range. ^a^Contrast agent was recorded within one month before surgery. ^b^ΔScr was calculated as difference between serum creatinine within 48 h before surgery and baseline.

### Primary outcome

**Figure 1:**
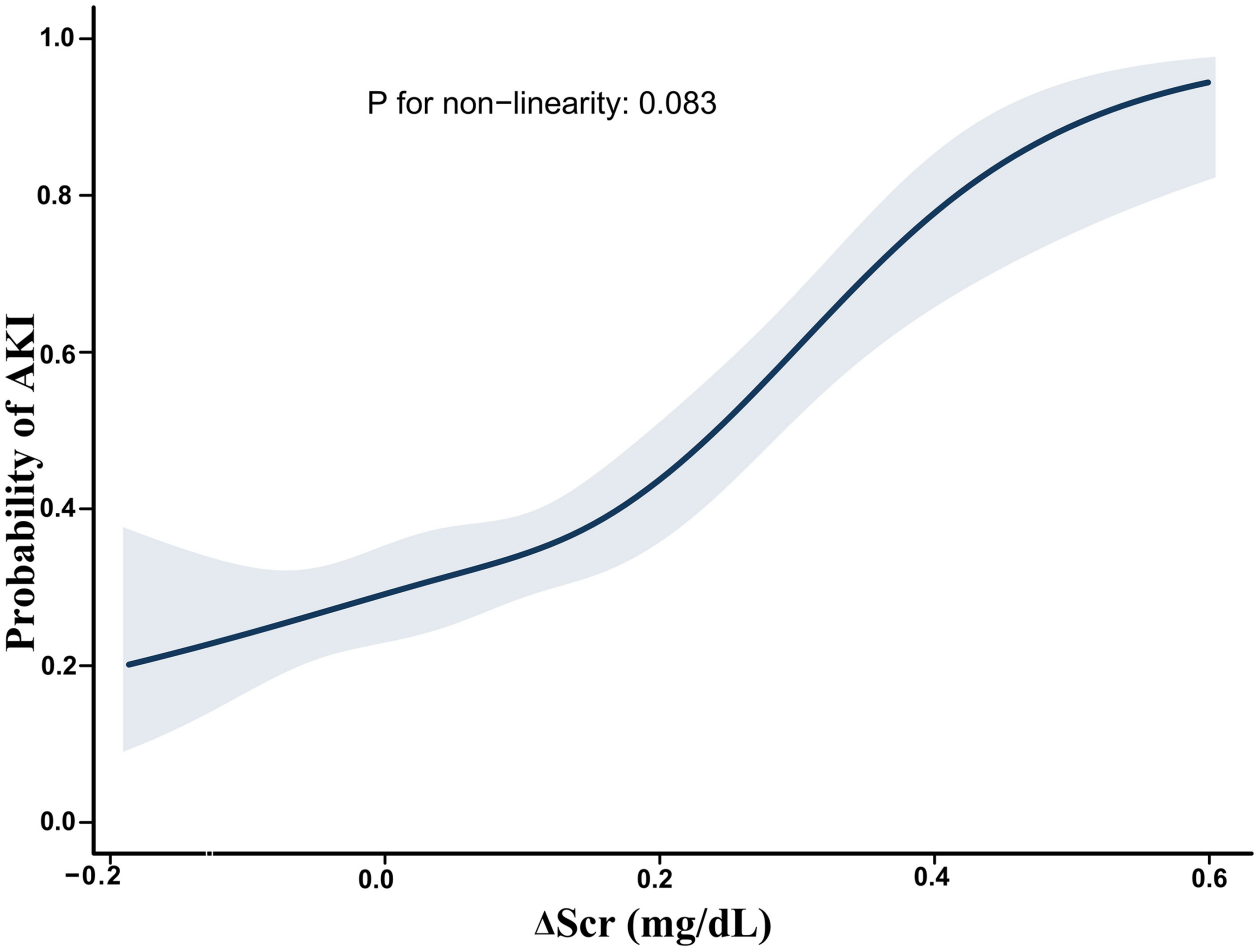
Estimated probability of acute kidney injury development. Restricted cubic spline curves were used to assess the nonlinear associations between ΔScr and AKI development. Shaded area represents 95% CIs. ΔScr indicates change in preoperative serum creatinine (calculated as difference between serum creatinine within 48 h before surgery and baseline).

Of the 560 patients included in the study, 225 (40.2%) developed AKI after surgery, 57 (10.2%) developed severe AKI, and 64 (11.4%) experienced AKI non-recovery within 7 days of AKI onset. The estimated probabilities of AKI, severe AKI, and AKI non-recovery increased linearly with rising ΔScr (Figure 1; Supplementary Figures S3, S4). Multivariable logistic regression models using Δscr as a continuous variable demonstrated a significant association between ΔScr and the risk of AKI, severe AKI, and AKI non-recovery. Each 0.1 mg/dl increase in ΔScr was associated with a higher risk of AKI [adjusted odds ratio (AOR), 1.51; 95% confidence interval (CI), 1.32–1.72], severe AKI (AOR, 1.45; 95% CI, 1.24–1.70), and AKI non-recovery (AOR, 1.37; 95% CI, 1.19–1.59) (Supplementary Table S1). When patients were grouped into three categories, elevated ΔScr was associated with an increased risk of AKI (AOR, 4.94; 95% CI, 2.50–9.77), severe AKI (AOR, 5.05; 95% CI, 2.35–10.85), and AKI non-recovery (AOR, 3.21; 95% CI, 1.57–6.54) compared with normal ΔScr. In contrast, negative ΔScr was associated with a lower risk of AKI (AOR, 0.52; 95% CI, 0.31–0.88); however, no significant association was observed with severe AKI or AKI non-recovery (Table 2).

**Table 2:** Association between ΔScr group and postoperative AKI.

**Primary outcome**	**Negative ΔScr (*n* = 127)**	**Normal ΔScr (*n* = 358)**	**Elevated ΔScr (*n* = 75)**
**AKI (KDIGO Stage 1 or 2 or 3)**			
Participants, No. (%)	32 (25.2)	132 (36.9)	61 (81.3)
Adjusted OR (95% CI)^a^	0.52 (0.31–0.88)	1.00 (Reference)	4.94 (2.50–9.77)
**Severe AKI (KDIGO Stage 2 or 3)**			
Participants, No. (%)	6 (4.7)	26 (7.3)	25 (33.3)
Adjusted OR (95% CI)^a^	0.53 (0.18–1.62)	1.00 (Reference)	5.05 (2.35–10.85)
**AKI non-recovery**			
Participants, No. (%)	9 (7.1)	32 (8.9)	23 (30.7)
Adjusted OR (95% CI)^a^	0.84 (0.35–2.00)	1.00 (Reference)	3.21 (1.57–6.54)

AKI, acute kidney injury; CI, confidence interval; OR, odds ratio. ^a^Adjusted for age, gender, baseline eGFR, hypertension, diabetes mellitus, EuroSCORE II, NYHA class, contrast agents, ACEIs or ARBs, diuretics, statins, NSAIDs, cardiopulmonary bypass, type of surgery, surgery time, and intraoperative blood transfusion volume.

### Secondary outcomes

**Figure 2:**
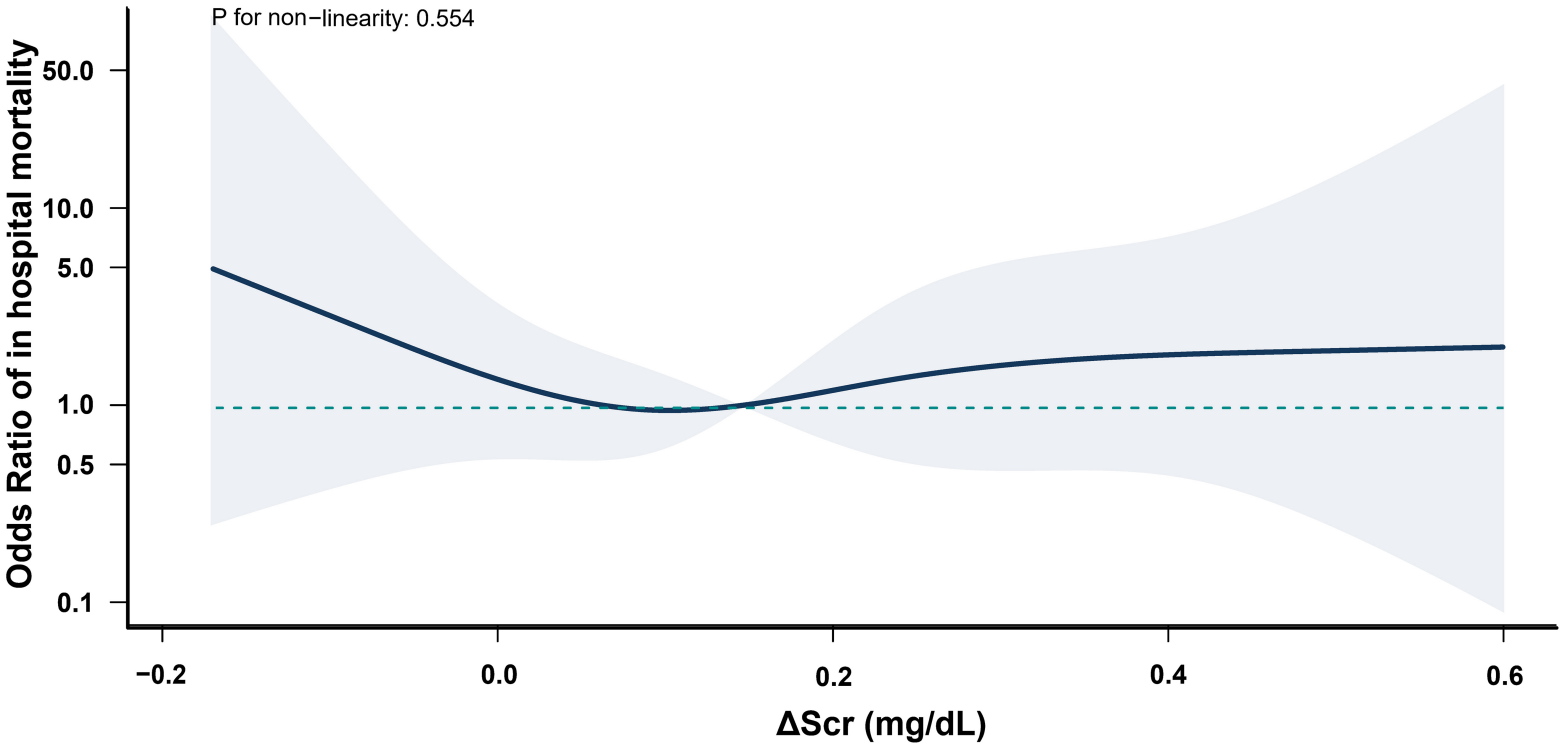
Association between ΔScr and in-hospital mortality in postoperative AKI patients. All patients who died during hospitalization had developed AKI. ΔScr represents the change in preoperative serum creatinine (calculated as difference between the serum creatinine within 48 h before surgery and baseline). Shaded area indicates 95% CIs, and while the solid line represents the adjusted odds ratios for in-hospital mortality across ΔScr values.

**Figure 3:**
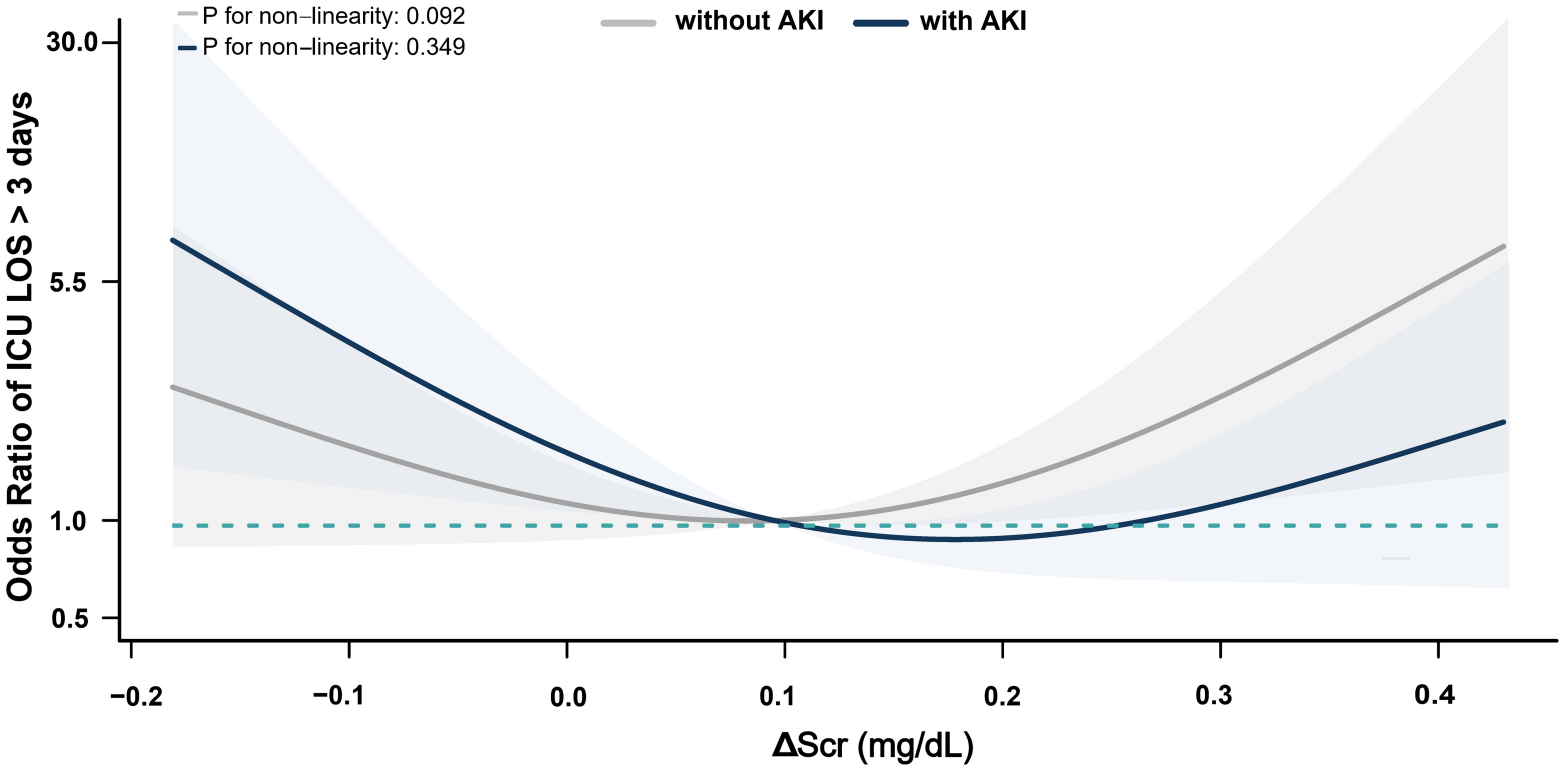
Association between ΔScr and ICU LOS >72 h in Patients with postoperative AKI. ΔScr represents the change in preoperative serum creatinine (calculated as difference between serum creatinine within 48 h before surgery and baseline). Shaded area indicates 95% CIs, and while the solid line represents the adjusted odds ratios for ICU LOS >72 h across ΔScr values.

Multivariable logistic regression models revealed no significant association between ΔScr and the risk of in-hospital mortality (AOR, 0.81; 95% CI, 0.63–1.06) or ICU LOS >72 h (AOR, 1.07; 95% CI, 0.96–1.19) when ΔScr was treated as a continuous variable, with each 0.1 mg/dl increase (Supplementary Tables S2, S3). Secondary outcomes were further analyzed based on the presence of postoperative AKI. All patients who died during hospitalization had developed AKI. In AKI patients, negative ΔScr was associated with higher rates of in-hospital mortality and prolonged ICU LOS compared to non-AKI patients, while elevated ΔScr did not demonstrate significant differences (Supplementary Table S4). Furthermore, in these patients, negative ΔScr increased the risk of in-hospital mortality (AOR, 4.50; 95% CI, 1.00–20.15) and ICU LOS >72 h (AOR, 2.81; 95% CI, 1.13–6.96) compared to normal ΔScr; however, elevated ΔScr was not associated with these outcomes. In contrast, in non-AKI patients, neither negative nor elevated ΔScr was associated with in-hospital mortality or prolonged ICU LOS (Table 3). Restricted cubic spline curves demonstrated similar results when ΔScr was treated as a continuous variable (Figures 2, 3).

**Table 3:** Association between ΔScr group and secondary outcomes.

**Secondary outcome**	**AKI status**	**Negative ΔScr**	**Normal ΔScr**	**Elevated ΔScr**
**In-hospital mortality**				
Adjusted OR (95% CI)^*a*^	With AKI	4.50 (1.00–20.15)	1 (reference)	0.84 (0.20–3.57)
	Without AKI	NA^*^	1 (reference)	NA^*^
**ICU LOS** > **72 h**				
Adjusted OR (95% CI)^*a*^	With AKI	2.81 (1.13–6.96)	1 (reference)	1.76 (0.86–3.61)
	Without AKI	1.19 (0.61–2.32)	1 (reference)	1.64 (0.43–6.19)

AKI, acute kidney injury; CI, confidence interval; ICU LOS, intensive care unit length of stay; OR, odds ratio. ^*a*^Adjusted for age, gender, baseline eGFR, hypertension, diabetes mellitus, EuroSCORE, NYHA class, contrast agents, ACEIs or ARBs, diuretics, statins, NSAIDs, cardiopulmonary bypass, type of surgery, surgery time, and intraoperative blood transfusion volume. ^*^The analysis could not be performed due to insufficient events.

### Sensitivity analysis

After excluding patients with serum creatinine ≥1.5 times baseline within 48 h before surgery, elevated ΔScr was associated with an increased risk of AKI (AOR, 4.23; 95% CI, 1.81–9.84), severe AKI (AOR, 4.32; 95% CI, 1.76–10.64), and AKI non-recovery (AOR, 2.69; 95% CI, 1.14–6.37; Supplementary Table S5). Negative ΔScr remained associated with higher in-hospital mortality (AOR 4.75; 95% CI 1.41–16.02) and ICU LOS >72 h (AOR 2.93; 95% CI 1.25–6.89) in postoperative AKI patients (Supplementary Table S6). These findings were consistent with the primary analysis.

## Discussion

In this study, we demonstrated that changes in preoperative serum creatinine from baseline were associated with cardiac surgery-associated AKI (CSA-AKI). Elevated ΔScr was associated with an increased risk of AKI, severe AKI and AKI non-recovery compared with normal ΔScr, while negative ΔScr was not associated with an increased risk. Moreover, when combined with postoperative AKI, these changes provided prognostic value. Negative changes in serum creatinine were associated with a higher risk of in-hospital mortality and prolonged ICU LOS in AKI patients, while elevated changes showed no such association. Additionally, neither negative nor elevated ΔScr was associated with these outcomes in non-AKI patients.

Previous research has demonstrated that preoperative kidney dysfunction is associated with an increased risk of AKI, elevated preoperative serum creatinine and declining glomerular filtration rate are associated with a higher likelihood of AKI development (24–26). Additionally, changes in serum creatinine levels offer valuable insights into AKI risk assessment. Ho et al. (27) demonstrated that small fluctuations in serum creatinine levels shortly after surgery can differentiate between low and high AKI risk. Similarly, Karkouti et al. (28) observed that an early postoperative rise in serum creatinine was linked to AKI following cardiac surgery, with an area under the receiver operating characteristic curve of 0.7. Building on these findings, our study observed that preoperative changes in serum creatinine from baseline were also associated with an increased risk of AKI. Specifically, for each 0.1 mg/dl increase in preoperative serum creatinine, the likelihood of developing AKI, severe AKI, and AKI non-recovery rose by 51%, 45%, and 37%, respectively. These results provide valuable insights into pre-surgery changes in serum creatinine from baseline for evaluating the risk of AKI.

AKI is a serious complication of cardiac surgery, associated with increased morbidity and mortality. A comprehensive meta-analysis of 91 studies revealed that the mortality rate for CSA-AKI patients was 10.6%, significantly higher than the 1.4% observed in patients without AKI. The pooled odds ratio for mortality associated with AKI was 7.0. Moreover, CSA-AKI patients had prolonged ICU LOS, with an average of 5.4 days compared to 2.2 days for those without AKI (29). Consistent with previous studies, our study also showed that the of AKI was associated with higher mortality and prolonged ICU LOS. Notably, the relationship between AKI and mortality varied across patterns of preoperative serum creatinine change.

In our study, negative changes in preoperative serum creatinine were associated with a lower risk of postoperative acute kidney injury (AKI) but a higher risk of in-hospital mortality and prolonged ICU stay among patients who developed AKI. This seemingly paradoxical finding may be explained by the fact that serum creatinine levels are affected not only by kidney function but also by muscle mass and metabolic state. As a byproduct of muscle metabolism, serum creatinine tends to be lower in individuals with reduced muscle mass. loss of muscle mass can result from inflammation, reduced food intake, and decreased physical activity during the preoperative period (30, 31). Additional factors that contributing to decreased serum creatinine production include female sex and advanced age. Conversely, increased creatinine clearance, which lowers serum creatinine levels, can occur in conditions such as pregnancy and augmented renal clearance (12, 13, 32). Furthermore, fluid overload and enhanced renal filtration may further reduce serum creatinine concentrations in critically ill patients (33, 34).

Building on these findings, our study further suggests that loss of muscle mass was likely the primary contributor to negative changes in preoperative serum creatinine, which were linked to a lower risk of AKI. To illustrate this effect, consider a patient with a baseline serum creatinine of 1.0 mg/dl. A postoperative increase to 1.5 mg/dl constitutes a 0.5 mg/dl rise, meeting the diagnostic threshold for AKI. If muscle wasting lowers preoperative serum creatinine to 0.9 mg/dl, a postoperative increase to 1.5 mg/dl would then require a 0.6 mg/dl rise. By contrast, if 0.9 mg/dl is established as the baseline, an increase of only 0.45 mg/dl would meet the AKI diagnostic criteria. This discrepancy may contribute to a lower observed incidence of AKI in patients with negative changes in preoperative serum creatinine. Furthermore, when AKI is diagnosed in these patients, it may indicate more severe kidney injury and a worse overall clinical condition. This could explain the association between negative preoperative serum creatinine changes and higher in-hospital mortality and prolonged ICU LOS among AKI patients. Our findings are consistent with prior evidence that reduced muscle mass is associated with adverse clinical outcomes (32, 35–37). In contrast, no significant association was observed between elevated changes in preoperative creatinine and poor outcomes. A possible explanation is that significant elevation in preoperative creatinine from baseline was rare, with an average increase of merely 0.4 mg/dl.

Our study has several strengths. Serum creatinine is a widely used marker for monitoring renal function, and its elevations are traditionally regarded as indicators of renal impairment. Our findings suggest that changes in serum creatinine may also be clinically relevant for assessing AKI risk and prognosis in cardiac surgery patients. This underscores the need for dynamic monitoring of serum creatinine during preoperative assessment rather than focusing solely on elevations. Moreover, preoperative creatinine changes expand on the role of biomarkers in early risk stratification. Prior research has demonstrated that preoperative biomarkers are associated with the risk of cardiac surgery-associated AKI (38). A recent study identified elevated preoperative neopterin levels as an independent predictor of AKI in patients undergoing on-pump cardiac surgery (39). These findings suggest potential approaches to incorporating diverse biological markers into preoperative evaluation to enhance risk assessment and optimize perioperative management. Furthermore, our study revealed that negative changes in preoperative serum creatinine were associated with higher in-hospital mortality and prolonged ICU LOS among cardiac surgery patients who developed postoperative AKI. Such reductions may serve as early markers of poor prognosis, particularly in high-risk cardiac surgical populations. Given the limited sample size, particularly the small number of patients with significant preoperative creatinine decreases, further validation in larger, multicenter cohorts is needed.

Our study also has several limitations. First, the retrospective design inherently limits causal inference. As exposures and outcomes were neither assigned nor measured prospectively, the observed associations may be subject to bias. In addition, although we adjusted for multiple confounders, certain relevant variables, such as intraoperative hemodynamics, fluid balance, and nephrotoxic drug use, were not measured and may have influenced the observed associations. Second, the selection of baseline serum creatinine may introduce bias (15, 40). Serum creatinine is an indirect marker of glomerular filtration, and its concentration can be influenced by non-renal factors such as volume status, muscle mass, and metabolic state. Currently, no universally accepted definition exists, and studies have variably used values obtained on the day of surgery or within 3–12 months prior to admission. Ideally, baseline creatinine should reflect stable kidney function based on multiple outpatient measurements, but such data are often unavailable in clinical practice. Consequently, patients with transient acute elevations may have been misclassified as having chronic kidney dysfunction at the time baseline creatinine was defined, leading to their exclusion. This potential misclassification may have affected the robustness of our findings. Third, the association between negative changes in preoperative creatinine and adverse outcomes may be partly explained by reduced muscle mass; however, our study lacked direct measures of muscle quantity (e.g., imaging or bioimpedance), which limits the strength of this interpretation. Additional contributors, such as perioperative fluid overload and measurement variability, may also compromise the robustness of our findings. Future studies incorporating objective assessments of muscle mass and fluid status are needed to clarify the underlying mechanisms. Finally, while we demonstrated the short-term implications of AKI in patients across different serum creatinine change categories before surgery, we could not assess long-term outcomes, such as the risk of progression to chronic kidney disease.

## Conclusion

This study highlights the preoperative serum creatinine changes provides clinically meaningful information. An upward trend is associated with increased risk of AKI development, while a negative ΔSCr is linked to higher in-hospital mortality and prolonged ICU LOS among patients who develop AKI. Recognizing these patterns may enhance preoperative risk stratification and guide individualized kidney protective strategies.

## Data Availability

The raw data supporting the conclusions of this article will be made available by the authors, without undue reservation.
