# Supplementary material for: Preoperative serum creatinine changes and acute kidney injury in patients underwent cardiac surgery
Source: Front Med (Lausanne). 2026 Jan 8;12:1584418. doi: 10.3389/fmed.2025.1584418 (PMC12824009; doi:10.3389/fmed.2025.1584418)
Supplement: Supplementary file 1 [file Data_Sheet_1.docx]

**Supplementary material for: Preoperative Serum Creatinine Changes and Acute Kidney Injury in Patients Underwent Cardiac Surgery**

Figure S1. Flowchart of inclusion and exclusion

Figure S2. Distribution of ΔScr

Figure S3. Estimated probability of severe AKI

Figure S4. Estimated probability of AKI non‒recovery

Table S1. Multivariable logistic regression applied to the primary outcome

Table S2**.** Multivariable logistic regression applied to the secondary outcome of in-hospital mortality

Table S3. Summary of multivariable logistic regression applied to the secondary outcome of ICU LOS >72 hours

Table S4. Secondary outcomes of AKI according to ΔScr

Table S5. Association between ΔScr group and postoperative AKI after excluding patients with serum creatinine ≥1.5 times baseline within 48 hours before surgery

Table S6. Association between ΔScr group and secondary outcomes after excluding patients with serum creatinine ≥1.5 times baseline within 48 hours before surgery


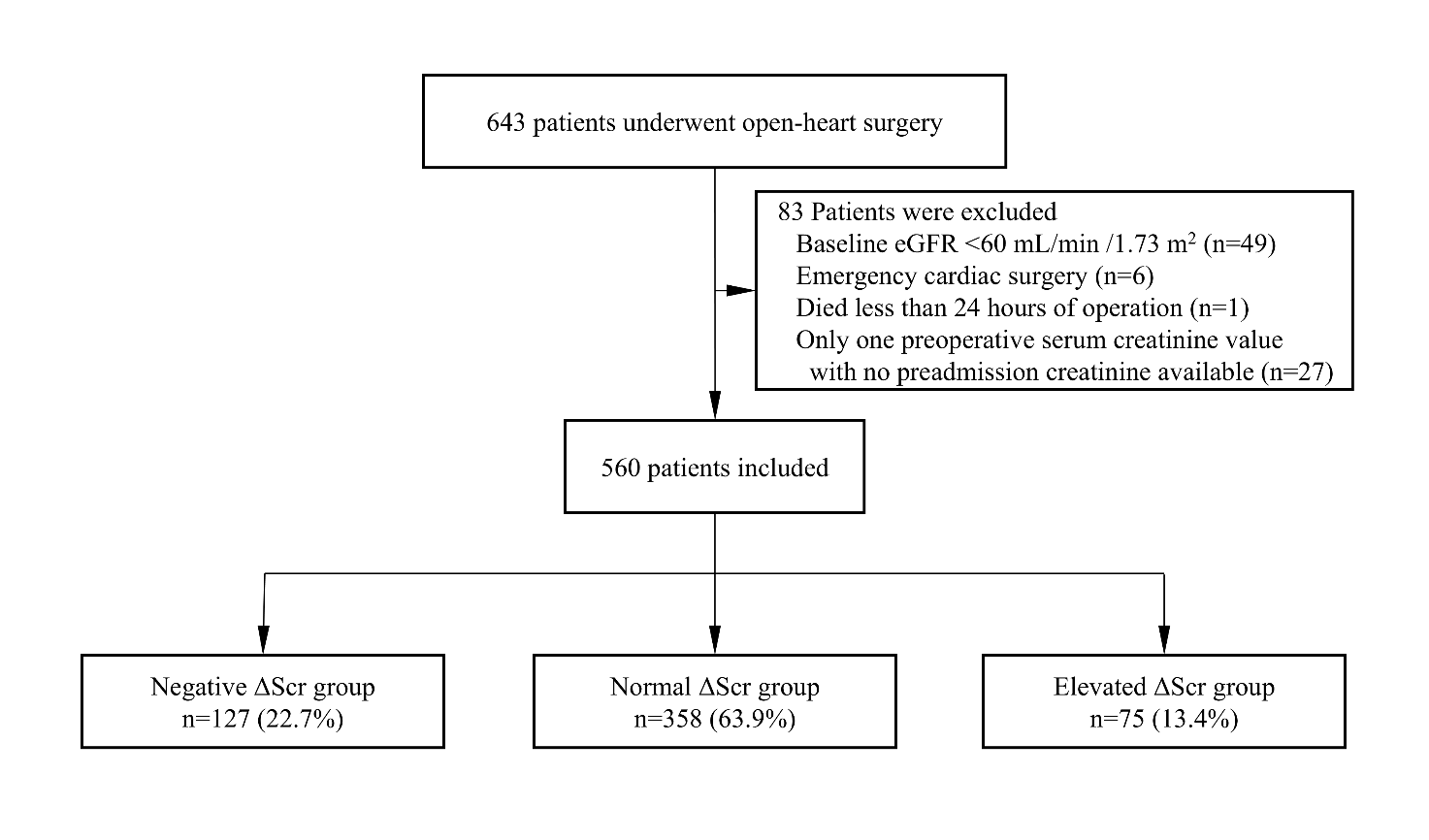


Figure S1. Flowchart of Inclusion and Exclusion. ΔScr indicates the change in preoperative serum creatinine (calculated as difference between the serum creatinine within 48 hours before surgery and baseline). eGFR, estimated glomerular filtration rate.


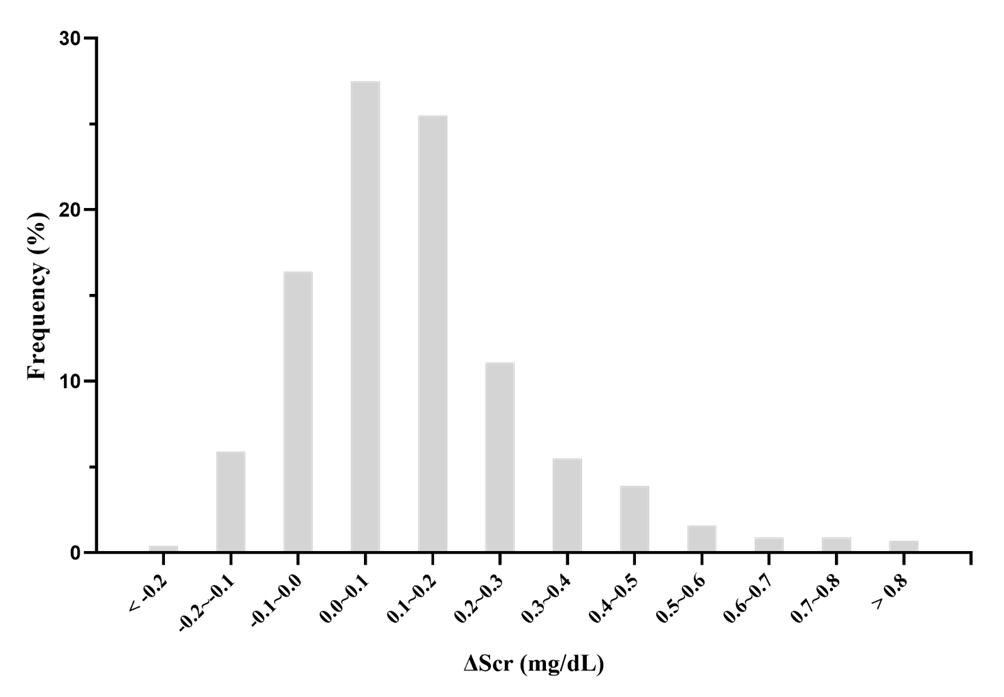


Figure S2. Distribution of ΔScr. ΔScr indicates change in preoperative serum creatinine (calculated as difference between the serum creatinine within 48 hours before surgery and baseline).


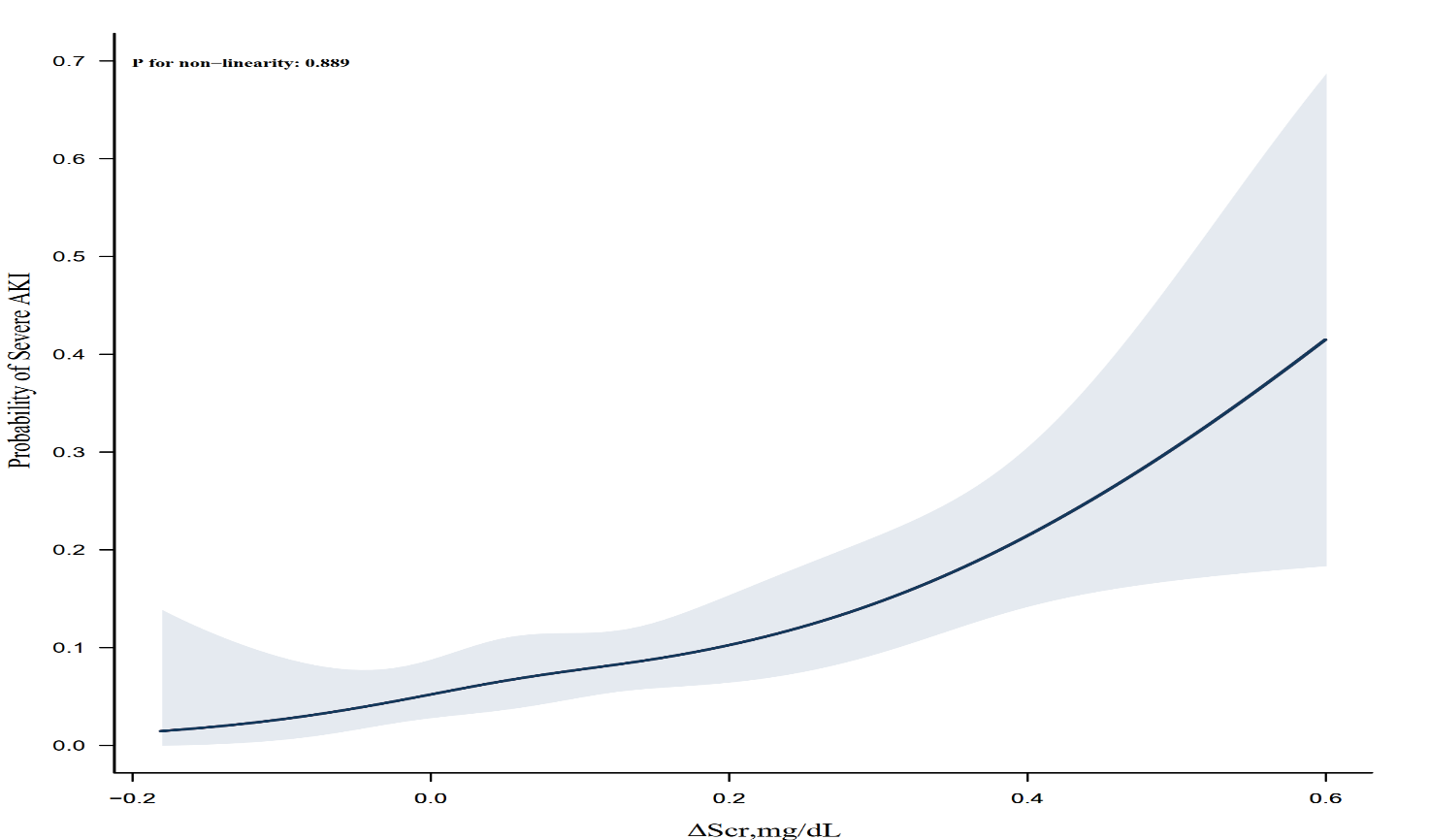


Figure S3. Estimated probability of severe AKI. Shaded area represents 95% CIs. ΔScr indicates change in preoperative serum creatinine (calculated as difference between serum creatinine within 48 hours before surgery and baseline).


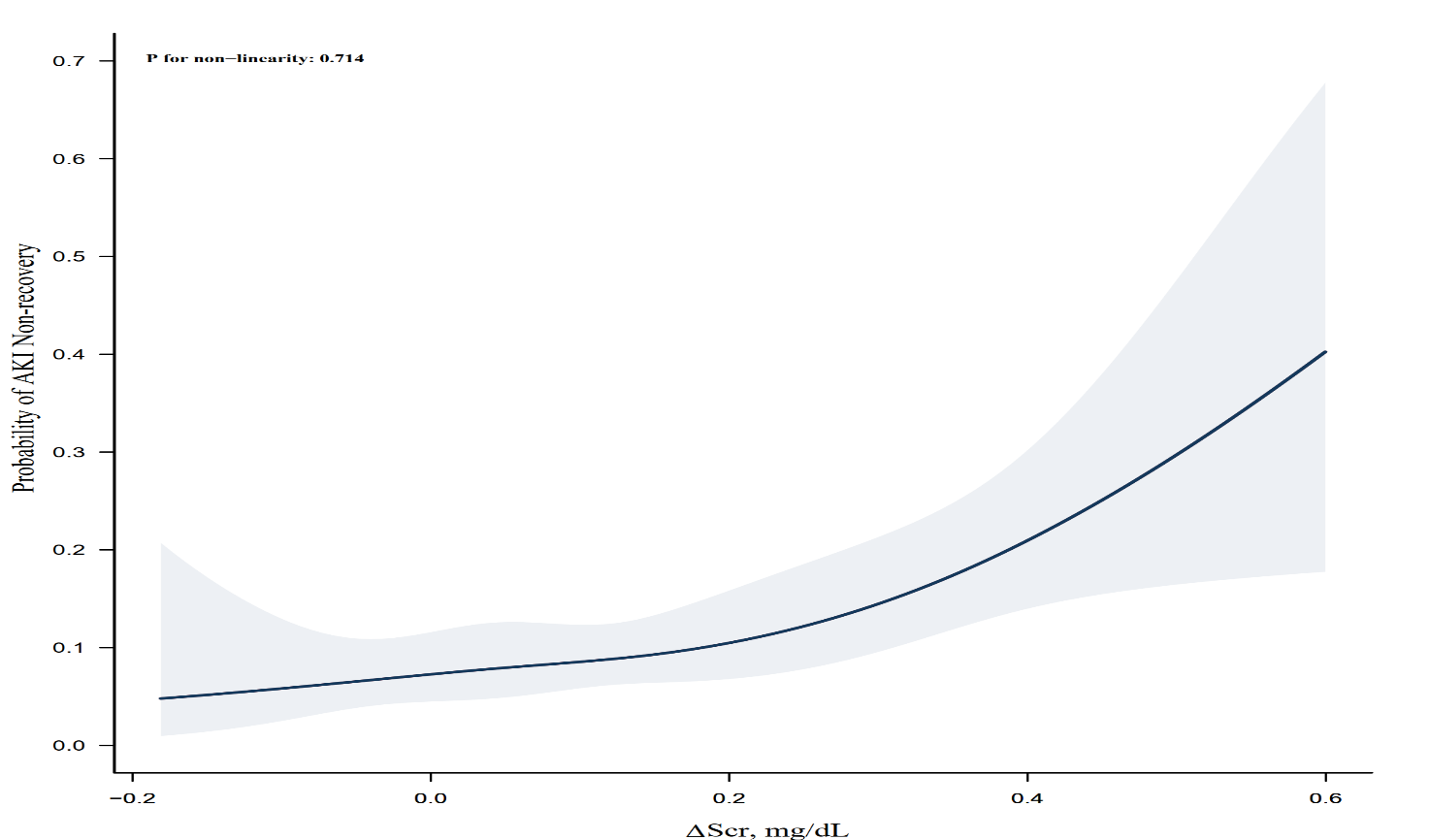


Figure S4. Estimated probability of AKI non‒recovery**.** Shaded area represents 95% CIs. ΔScr indicates change in preoperative serum creatinine (calculated as difference between serum creatinine within 48 hours before surgery and baseline).

Table S1. Multivariable logistic regression applied to the primary outcome

| Variable | Unadjusted OR (95%CI) | P-value | Adjusted OR (95%CI) | P-value |
| --- | --- | --- | --- | --- |
| AKI (KDIGO Stage 1 or 2 or 3) | | | | |
| ΔScr, per 0.1mg/dL | 1.56 (1.39–1.76) | < 0.001 | 1.51 (1.32–1.72) | < 0.001 |
| baseline eGFR, mL/min /1.73 m^2^ | 0.97 (0.96–0.99) | <0.001 | 0.99 (0.97–1.01) | 0.167 |
| Age, year | 1.01 (0.99–1.03) | 0.215 | 0.97 (0.94–1.01) | 0.112 |
| Euroscore II | 1.31 (1.19–1.46) | <0.001 | 1.40 (1.17–1.68) | <0.001 |
| Male | 1.11 (0.78–1.58) | 0.547 | 1.51 (0.96–2.38) | 0.072 |
| Contrast agent | 1.09 (0.68–1.75) | 0.710 | 1.67 (0.84–3.34) | 0.147 |
| Hypertension | 1.17 (0.83–1.66) | 0.361 | 1.11 (0.70–1.76) | 0.648 |
| Diabetes mellitus | 0.79 (0.54–1.15) | 0.219 | 1.21 (0.74–1.98) | 0.452 |
| Stroke | 0.80 (0.48–1.32) | 0.374 | 0.63 (0.34–1.16) | 0.140 |
| NYHA class III/IV | 2.24 (1.55–3.23) | <0.001 | 1.40 (0.85–2.31) | 0.185 |
| Type of surgery |  |  |  |  |
| Coronary artery bypass graft | 1.00 (Reference) |  | 1.00 (Reference) |  |
| Valve | 2.48 (1.71–3.60) | <0.001 | 2.52 (1.00–6.33) | 0.049 |
| Combined | 5.26 (2.71–10.2) | <0.001 | 0.96 (0.32–2.90) | 0.937 |
| Other | 0.24 (0.05–1.05) | 0.057 | 0.71 (0.13–3.84) | 0.695 |
| Cardiopulmonary bypass | 3.00 (2.10–4.28) | <0.001 | 1.38 (0.58–3.28) | 0.462 |
| Statins | 1.18 (0.84–1.66) | 0.326 | 0.76 (0.39–1.50) | 0.434 |
| ACEIs or ARBs | 0.88 (0.62–1.23) | 0.440 | 0.76 (0.50–1.16) | 0.208 |
| NSAIDs | 1.34 (0.96–1.88) | 0.088 | 1.79 (0.90–3.55) | 0.095 |
| Diuretics | 0.83 (0.59–1.17) | 0.294 | 0.87 (0.53–1.43) | 0.570 |
| Surgery time, per 30 min | 1.26 (1.18–1.34) | <0.001 | 1.25 (1.14–1.36) | <0.001 |
| Intraoperative blood product transfusion, per 100 mL | 1.07 (1.04–1.09) | <0.001 | 0.98 (0.94–1.01) | 0.203 |
| Severe AKI (KDIGO Stage 2 or 3) | | | | |
| ΔScr, per 0.1mg/dL | 1.51 (1.32–1.73) | <0.001 | 1.45 (1.24–1.70) | <0.001 |
| baseline eGFR, mL/min /1.73 m^2^ | 0.98 (0.96–1.00) | 0.063 | 0.99 (0.96–1.02) | 0.563 |
| Age, year | 0.99 (0.97–1.02) | 0.575 | 0.95 (0.90–0.99) | 0.018 |
| Euroscore | 1.36 (1.18–1.57) | <0.001 | 1.62 (1.25–2.08) | <0.001 |
| Male | 0.71 (0.41–1.24) | 0.232 | 0.79 (0.38–1.64) | 0.531 |
| Contrast agent | 0.57 (0.30–1.12) | 0.103 | 0.76 (0.27–2.14) | 0.597 |
| Hypertension | 1.54 (0.86–2.77) | 0.149 | 1.93 (0.88–4.23) | 0.102 |
| Diabetes mellitus | 0.95 (0.51–1.74) | 0.856 | 1.48 (0.65–3.38) | 0.354 |
| Stroke | 1.22 (0.57–2.60) | 0.606 | 0.99 (0.37–2.66) | 0.991 |
| NYHA class III/IV | 2.24 (1.29–3.91) | 0.004 | 1.58 (0.74–3.39) | 0.238 |
| Type of surgery |  |  |  |  |
| Coronary artery bypass graft | 1.00 (Reference) |  | 1.00 (Reference) |  |
| Valve | 3.23 (1.71–6.13) | <0.001 | 1.27 (0.33–4.88) | 0.727 |
| Combined | 4.05 (1.66–9.87) | 0.002 | 0.20 (0.04–0.95) | 0.043 |
| Other | NA^*^ | NA^*^ | NA^*^ | NA^*^ |
| Cardiopulmonary bypass | 5.31 (2.55–11.06) | <0.001 | 3.63 (1.02–12.84) | 0.046 |
| Statins | 1.20 (0.69–2.07) | 0.521 | 0.51 (0.17–1.54) | 0.233 |
| ACEIs or ARBs | 1.17 (0.68–2.03) | 0.571 | 1.26 (0.63–2.54) | 0.511 |
| NSAIDs | 1.49 (0.86–2.6) | 0.155 | 2.98 (0.95–9.36) | 0.062 |
| Diuretics, | 0.87 (0.50–1.52) | 0.631 | 1.17 (0.5–2.75) | 0.710 |
| Surgery time, per 30 min | 1.29 (1.20–1.38) | <0.001 | 1.32 (1.15–1.52) | <0.001 |
| Intraoperative blood product transfusion, per 100 mL | 1.07 (1.05–1.10) | <0.001 | 0.97 (0.92–1.03) | 0.318 |
| AKI non-recovery | | | | |
| Scr, per 0.1mg/dL | 1.44 (1.27–1.64) | <0.001 | 1.37 (1.19–1.59) | <0.001 |
| baseline eGFR, mL/min /1.73 m^2^ | 0.99 (0.97–1.01) | 0.401 | 1.01 (0.99–1.04) | 0.301 |
| Age, year | 1.01 (0.98–1.04) | 0.505 | 0.99 (0.95–1.03) | 0.658 |
| Euroscore | 1.30 (1.13–1.49) | <0.001 | 1.40 (1.12–1.75) | 0.003 |
| Male | 1.21 (0.69–2.10) | 0.504 | 1.47 (0.75–2.86) | 0.262 |
| Contrast agent | 0.86 (0.43–1.72) | 0.666 | 1.25 (0.45–3.49) | 0.673 |
| Hypertension | 1.58 (0.90–2.75) | 0.110 | 1.64 (0.82–3.30) | 0.164 |
| Diabetes mellitus | 1.03 (0.58–1.83) | 0.914 | 1.49 (0.74–3.01) | 0.268 |
| Stroke | 1.05 (0.49–2.22) | 0.903 | 0.84 (0.35–2.03) | 0.698 |
| NYHA class III/IV | 1.92 (1.13–3.26) | 0.016 | 1.41 (0.70–2.83) | 0.336 |
| Type of surgery |  |  |  |  |
| Coronary artery bypass graft | 1.00 (Reference) |  | 1.00 (Reference) |  |
| Valve | 1.97 (1.12–3.48) | 0.019 | 2.32 (0.66–8.20) | 0.191 |
| Combined | 2.56 (1.10–5.93) | 0.029 | 0.43 (0.10–1.83) | 0.256 |
| Other | NA^*^ | NA^*^ | NA^*^ | NA^*^ |
| Cardiopulmonary bypass | 2.43 (1.37–4.32) | 0.002 | 1.09 (0.35–3.42) | 0.887 |
| Statins | 0.98 (0.58–1.66) | 0.952 | 0.41 (0.15~1.13) | 0.086 |
| ACEIs or ARBs | 1.05 (0.62–1.77) | 0.849 | 1.02 (0.55–1.89) | 0.948 |
| NSAIDs | 1.21 (0.72–2.04) | 0.476 | 2.34 (0.85–6.45) | 0.101 |
| Diuretics | 0.94 (0.56–1.60) | 0.823 | 0.96 (0.46–1.99) | 0.909 |
| Surgery time, per 30 min | 1.26 (1.18–1.35) | <0.001 | 1.30 (1.14–1.47) | <0.001 |
| Intraoperative blood product transfusion, per 100 mL | 1.06 (1.03–1.08) | <0.001 | 0.98 (0.94–1.03) | 0.472 |

Abbreviations: EuroSCOREII, European System for Cardiac Operative Risk Evaluation II; ACEI, angiotensin-converting enzyme inhibitor; ARB, angiotensin receptor blocker; NYHA, New York Heart Association; eGFR, estimated glomerular filtration rate; NSAID, non-steroidal anti-inflammatory drugs; CI, confidence interval; OR, odds ratio.

^*^ The analysis could not be performed due to insufficient event

Table S2. Multivariable logistic regression applied to the secondary outcome of in-hospital mortality

| Variable | Unadjusted OR (95%CI) | | | | **P-value** | **Adjusted OR (95%CI)** | **P-value** |
| --- | --- | --- | --- | --- | --- | --- | --- |
| In-hospital mortality (Total) | | | | | | | |
| ΔScr, per 0.1mg/dL | 0.95 (0.75–1.22) | | 0.708 | | | 0.81 (0.63–1.06) | 0.121 |
| baseline eGFR, mL/min /1.73 m^2^ | 1.01 (0.97‒1.04) | | 0.765 | | | 1.02 (0.97‒1.07) | 0.438 |
| Age, year | 0.98 (0.94‒1.02) | | 0.391 | | | 0.96 (0.90‒1.03) | 0.307 |
| Euroscore | 1.42 (1.15‒1.75) | | 0.001 | | | 1.82 (1.28‒2.61) | 0.001 |
| Male | 1.16 (0.46‒2.93) | | 0.751 | | | 1.52 (0.45‒5.17) | 0.501 |
| Contrast agent | 0.76 (0.25‒2.32) | | 0.634 | | | 2.59 (0.38‒17.61) | 0.331 |
| Hypertension | 0.91 (0.38‒2.19) | | 0.825 | | | 1.13 (0.32‒3.92) | 0.852 |
| Diabetes mellitus | 0.56 (0.19‒1.7) | | 0.307 | | | 0.74 (0.18‒3.13) | 0.688 |
| Stroke | 1.06 (0.31‒3.7) | | 0.922 | | | 1.07 (0.19‒6.15) | 0.94 |
| NYHA class III/IV | 2.61 (1.09‒6.26) | | 0.032 | | | 2.71 (0.77‒9.55) | 0.121 |
| Type of surgery |  | |  | | |  |  |
| Coronary artery bypass graft | 1.00 (Reference) | |  | | | 1.00 (Reference) |  |
| Valve | 2.48 (0.9‒6.82) | | 0.078 | | | 4.35 (0.5‒37.65) | 0.181 |
| Combined | 4.12 (1.12‒15.19) | | 0.033 | | | 0.32 (0.03‒3.02) | 0.318 |
| Other | NA^*^ | | NA^*^ | | | NA^*^ | NA^*^ |
| Cardiopulmonary bypass | 3.84 (1.28‒11.58) | | 0.017 | | | 1.18 (0.16‒8.47) | 0.871 |
| Statins | 2.02 (0.80‒5.09) | | 0.135 | | | 0.44 (0.07‒2.63) | 0.366 |
| ACEIs or ARBs | 1.23 (0.52‒2.95) | | 0.637 | | | 0.69 (0.22‒2.17) | 0.531 |
| NSAIDs | 2.15 (0.85‒5.40) | | 0.105 | | | 6.85 (1.10‒42.69) | 0.039 |
| Diuretics | 0.92 (0.38‒2.22) | | 0.852 | | | 1.63 (0.46‒5.79) | 0.447 |
| Surgery time, per 30 min | 1.36 (1.23‒1.50) | | <0.001 | | | 1.76 (1.37‒2.25) | <0.001 |
| Intraoperative blood product transfusion, per 100 mL | 1.06 (1.03‒1.09) | | <0.001 | | | 1.11 (1.03‒1.19) | 0.005 |
| In-hospital mortality (with AKI) | | | | | | | |
| ΔScr, per 0.1mg/dL | | 0.77 (0.59~1.00) | | 0.047 | | 0.74 (0.57~0.98) | 0.035 |
| baseline eGFR, mL/min /1.73 m^2^ | | 1.02 (0.99‒1.06) | | 0.218 | | 1.02 (0.97‒1.07) | 0.516 |
| Age, year | | 0.98 (0.94‒1.02) | | 0.238 | | 0.98 (0.91‒1.06) | 0.598 |
| Euroscore | | 1.25 (1.00‒1.55) | | 0.046 | | 1.61 (1.1‒2.35) | 0.015 |
| Male | | 1.09 (0.42‒2.83) | | 0.858 | | 1.34 (0.35‒5.11) | 0.664 |
| Contrast agent | | 0.70 (0.22‒2.24) | | 0.553 | | 1.56 (0.2‒12.4) | 0.673 |
| Hypertension | | 0.81 (0.33‒2.01) | | 0.647 | | 1.13 (0.3‒4.34) | 0.855 |
| Diabetes mellitus | | 0.64 (0.21‒1.98) | | 0.436 | | 0.64 (0.14‒2.87) | 0.559 |
| Stroke | | 1.25 (0.34‒4.56) | | 0.735 | | 0.91 (0.13‒6.16) | 0.921 |
| NYHA class III/IV | | 1.67 (0.68‒4.11) | | 0.264 | | 2.05 (0.54‒7.76) | 0.293 |
| Type of surgery | |  | |  | |  |  |
| Coronary artery bypass graft | | 1.00 (Reference) | |  | | 1.00 (Reference) |  |
| Valve | | 1.44 (0.51‒4.06) | | 0.495 | | 3.22 (0.3‒34.3) | 0.333 |
| Combined | | 1.75 (0.46‒6.64) | | 0.413 | | 0.65 (0.06‒6.51) | 0.711 |
| Other | | NA^*^ | | NA^*^ | | NA^*^ | NA^*^ |
| Cardiopulmonary bypass | | 1.99 (0.64‒6.14) | | 0.233 | | 0.83 (0.11~6.5) | 0.857 |
| Statins | | 1.89 (0.73‒4.87) | | 0.19 | | 0.35 (0.04‒2.88) | 0.328 |
| ACEIs or ARBs | | 1.37 (0.56‒3.36) | | 0.497 | | 0.94 (0.28‒3.13) | 0.915 |
| NSAIDs | | 1.85 (0.72‒4.77) | | 0.204 | | 5.03 (0.58‒43.62) | 0.142 |
| Diuretics | | 1.03 (0.42‒2.56) | | 0.946 | | 1.20 (0.31‒4.62) | 0.791 |
| Surgery time, per 30 min | | 1.06 (1.02‒1.09) | | 0.003 | | 0.93 (0.86‒1.00) | 0.064 |
| Intraoperative blood product transfusion, per 100 mL | | 1.31 (1.17‒1.46) | | <0.001 | | 1.61 (1.24‒2.09) | <0.001 |

Abbreviations: EuroSCOREII, European System for Cardiac Operative Risk Evaluation II; ACEI, angiotensin-converting enzyme inhibitor; ARB, angiotensin receptor blocker; NYHA, New York Heart Association; eGFR, estimated glomerular filtration rate; NSAID, non-steroidal anti-inflammatory drugs; CI, confidence interval; OR, odds ratio; AKI, acute kidney injury.

* The analysis could not be performed due to insufficient event.

Table S3. Summary of multivariable logistic regression applied to the secondary outcome of ICU LOS >72 hours

| Variable | Unadjusted OR (95%CI) | | P-value | Adjusted OR (95%CI) | P-value |
| --- | --- | --- | --- | --- | --- |
| ICU LOS > 72 hours (Total) | | | | | |
| ΔScr, per 0.1mg/dL | 1.12 (1.02–1.23) | 0.015 | | 1.07 (0.96–1.19) | 0.204 |
| baseline eGFR, mL/min /1.73 m^2^ | 0.98 (0.97‒1.00) | 0.013 | | 0.99 (0.97‒1.01) | 0.445 |
| Age, year | 1.02 (1.00‒1.04) | 0.108 | | 0.99 (0.96‒1.03) | 0.743 |
| Euroscore | 1.19 (1.08‒1.32) | 0.001 | | 1.13 (0.96‒1.34) | 0.138 |
| Male | 1.11 (0.77‒1.62) | 0.573 | | 1.14 (0.73‒1.78) | 0.567 |
| Contrast agent | 1.17 (0.70‒1.95) | 0.540 | | 1.19 (0.59‒2.41) | 0.628 |
| Hypertension | 1.46 (1.01‒2.12) | 0.046 | | 1.25 (0.79‒1.98) | 0.339 |
| Diabetes mellitus | 0.85 (0.57‒1.26) | 0.413 | | 0.81 (0.5‒1.31) | 0.389 |
| Stroke | 1.79 (1.09‒2.93) | 0.022 | | 1.53 (0.88‒2.66) | 0.129 |
| NYHA class III/IV | 1.34 (0.91‒1.96) | 0.137 | | 1.33 (0.81‒2.19) | 0.257 |
| Type of surgery |  |  | |  |  |
| Coronary artery bypass graft | 1.00 (Reference) |  | | 1.00 (Reference) |  |
| Valve | 1.07 (0.73‒1.58) | 0.723 | | 1.14 (0.46‒2.81) | 0.782 |
| Combined | 1.25 (0.65‒2.37) | 0.505 | | 0.35 (0.12‒1.04) | 0.059 |
| Other | 0.23 (0.05‒0.99) | 0.049 | | 0.71 (0.14‒3.73) | 0.689 |
| Cardiopulmonary bypass | 1.26 (0.88‒1.81) | 0.208 | | 0.58 (0.25‒1.33) | 0.196 |
| Statins | 1.33 (0.92‒1.90) | 0.125 | | 0.91 (0.47‒1.76) | 0.781 |
| ACEIs or ARBs | 1.25 (0.87‒1.79) | 0.226 | | 1.28 (0.85‒1.93) | 0.24 |
| NSAIDs | 1.35 (0.94‒1.93) | 0.106 | | 1.13 (0.58‒2.20) | 0.71 |
| Diuretics | 0.69 (0.48‒1.00) | 0.047 | | 0.68 (0.42‒1.10) | 0.113 |
| Surgery time, per 30 min | 1.24 (1.16‒1.31) | <0.001 | | 1.25 (1.14‒1.36) | <0.001 |
| Intraoperative blood product transfusion, per 100 mL | 1.05 (1.03‒1.07) | <0.001 | | 1.02 (0.99‒1.05) | 0.28 |
| ICU LOS >72 hours (With AKI) | | | | | |
| ΔScr, per 0.1mg/dL | 0.97 (0.86–1.09) | 0.612 | | 0.97 (0.85–1.11) | 0.673 |
| baseline eGFR, mL/min /1.73 m^2^ | 1.00 (0.98‒1.02) | 0.805 | | 0.99 (0.96‒1.01) | 0.352 |
| Age, year | 1.00 (0.97‒1.02) | 0.802 | | 0.98 (0.93‒1.02) | 0.321 |
| Euroscore | 1.00 (0.87‒1.15) | 0.997 | | 0.99 (0.79‒1.25) | 0.951 |
| Male | 1.29 (0.74‒2.23) | 0.368 | | 1.32 (0.66‒2.67) | 0.434 |
| Contrast agent | 1.25 (0.59‒2.63) | 0.560 | | 1.92 (0.65‒5.64) | 0.238 |
| Hypertension | 1.41 (0.82‒2.42) | 0.215 | | 1.51 (0.75‒3.05) | 0.251 |
| Diabetes mellitus | 1.12 (0.62‒2.02) | 0.715 | | 0.87 (0.42‒1.81) | 0.715 |
| Stroke | 2.08 (0.91‒4.77) | 0.083 | | 2.57 (0.97‒6.83) | 0.058 |
| NYHA class III/IV | 0.84 (0.49‒1.43) | 0.525 | | 1.04 (0.52‒2.06) | 0.921 |
| Type of surgery |  |  | |  |  |
| Coronary artery bypass graft | 1.00 (Reference) |  | | 1.00 (Reference) |  |
| Valve | 0.63 (0.36‒1.13) | 0.122 | | 2.07 (0.53‒8.13) | 0.295 |
| Combined | 0.47 (0.20‒1.08) | 0.075 | | 0.64 (0.14‒2.81) | 0.552 |
| Other | NA^*^ | NA^*^ | | NA^*^ | NA^*^ |
| Cardiopulmonary bypass | 0.63 (0.36‒1.11) | 0.113 | | 0.25 (0.07‒0.90) | 0.035 |
| Statins | 1.15 (0.68‒1.94) | 0.604 | | 0.45 (0.16‒1.26) | 0.127 |
| ACEIs or ARBs | 1.33 (0.78‒2.25) | 0.290 | | 1.35 (0.73‒2.49) | 0.347 |
| NSAIDs | 1.59 (0.94‒2.70) | 0.084 | | 3.01 (1.00‒9.08) | 0.050 |
| Diuretics | 0.76 (0.45‒1.28) | 0.303 | | 0.96 (0.44‒2.10) | 0.926 |
| Surgery time, per 30 min | 1.02 (0.99‒1.05) | 0.134 | | 1.00 (0.95‒1.06) | 0.893 |
| Intraoperative blood product transfusion, per 100 mL | 1.15 (1.06‒1.25) | <0.001 | | 1.28 (1.11‒1.48) | 0.001 |
| ICU LOS >72 hours (Without AKI) | | | | | |
| ΔScr, per 0.1mg/dL | 1.12 (0.92–1.37) | 0.264 | | 1.09 (0.88–1.35) | 0.448 |
| baseline eGFR, mL/min /1.73 m^2^ | 0.98 (0.96‒1.00) | 0.066 | | 1.01 (0.98‒1.04) | 0.589 |
| Age, year | 1.03 (1.00‒1.07) | 0.033 | | 1.01 (0.96‒1.07) | 0.646 |
| Euroscore | 1.30 (1.10‒1.54) | 0.002 | | 1.19 (0.89‒1.59) | 0.238 |
| Male | 0.91 (0.52‒1.58) | 0.739 | | 1.01 (0.49‒2.08) | 0.977 |
| Contrast agent | 1.07 (0.50‒2.25) | 0.868 | | 0.61 (0.2‒1.85) | 0.384 |
| Hypertension | 1.46 (0.83‒2.56) | 0.185 | | 1.17 (0.58‒2.36) | 0.664 |
| Diabetes mellitus | 0.73 (0.40‒1.33) | 0.301 | | 0.67 (0.32‒1.42) | 0.300 |
| Stroke | 2.03 (1.03‒4.01) | 0.041 | | 1.60 (0.72‒3.53) | 0.250 |
| NYHA class III/IV | 1.41 (0.77‒2.58) | 0.268 | | 1.61 (0.71‒3.67) | 0.253 |
| Type of surgery |  |  | |  |  |
| Coronary artery bypass graft | 1.00 (Reference) |  | | 1.00 (Reference) |  |
| Valve | 0.99 (0.55‒1.78) | 0.965 | | 0.68 (0.16~2.84) | 0.596 |
| Combined | 1.95 (0.63‒6.03) | 0.246 | | 0.22 (0.03‒1.67) | 0.142 |
| Other | NA^*^ | NA^*^ | | NA^*^ | NA^*^ |
| Cardiopulmonary bypass | 1.22 (0.71‒2.1) | 0.466 | | 0.88 (0.24‒3.2) | 0.840 |
| Statins | 1.45 (0.84‒2.49) | 0.181 | | 2.28 (0.85‒6.14) | 0.101 |
| ACEIs or ARBs | 1.34 (0.78‒2.3) | 0.286 | | 1.29 (0.69~2.42) | 0.433 |
| NSAIDs | 0.97 (0.57‒1.67) | 0.925 | | 0.40 (0.15‒1.07) | 0.069 |
| Diuretics | 0.67 (0.39‒1.15) | 0.145 | | 0.72 (0.35‒1.47) | 0.363 |
| Surgery time, per 30 min | 1.23 (1.12‒1.36) | <0.001 | | 1.22 (1.05‒1.42) | 0.008 |
| Intraoperative blood product transfusion, per 100 mL | 1.04 (1.01‒1.07) | 0.004 | | 1.03 (0.98‒1.08) | 0.255 |

Abbreviations: EuroSCOREII, European System for Cardiac Operative Risk Evaluation II; ACEI, angiotensin-converting enzyme inhibitor; ARB, angiotensin receptor blocker; NYHA, New York Heart Association; eGFR, estimated glomerular filtration rate; NSAID, non-steroidal anti-inflammatory drugs; CI, confidence interval; OR, odds ratio. AKI, acute kidney injury; ICU LOS, intensive care unit length of stay.

^*^The analysis could not be performed due to insufficient events.

Table S4. Secondary outcomes of AKI according to ΔScr

| Secondary outcome | ALL | Negative (n=127) | Normal (n=358) | Elevated (n=75) |
| --- | --- | --- | --- | --- |
| **In-hospital mortality, n (%)** | 21 (3.8) | 7 (5.5) | 10 (2.9) | 4 (5.3) |
| With AKI, n (%) | 21 (9.3) | 7/32 (21.9) | 10/132 (7.6) | 4/61 (6.5) |
| Without AKI, n (%) | 0 (0.0) | 0/95 (0) | 0/226 (0) | 0/14 (0) |
| P-value | <0.001 | <0.001 | <0.001 | 0.325 |
| **ICU LOS >72hours, n (%)** | 171 (30.5) | 40 (31.5) | 95 (26.5) | 36 (48) |
| With AKI, n (%) | 106 (47.1) | 21/32 (65.6) | 54/132 (40.6) | 31/61 (50.8) |
| Without AKI, n (%) | 66 (19.7) | 20/95 (21.1) | 41/226 (18.1) | 5/14 (35.7) |
| P-value | <0.001 | <0.001 | <0.001 | 0.308 |

Abbreviations: AKI, acute kidney injury; ICU LOS, intensive care unit length of stay

Table S5. Association between ΔScr group and postoperative AKI after excluding patients with serum creatinine ≥1.5 times baseline within 48 hours before surgery

| Primary Outcome | Negative ΔScr (n=127) | Normal ΔScr (n=355) | Elevated ΔScr (n=38) |
| --- | --- | --- | --- |
| AKI (KDIGO Stage 1 or 2 or 3) |  |  |  |
| Participants, No. (%) | 32 (25.2) | 131 (36.9) | 29 (76.3) |
| Adjusted OR (95% CI) ^a^ | 0.54 (0.33–0.89) | 1.00 (Reference) | 4.23 (1.81–9.84) |
| Severe AKI (KDIGO Stage 2 or 3) |  |  |  |
| Participants, No. (%) | 6 (4.7) | 26 (7.3) | 11 (28.9) |
| Adjusted OR (95% CI) ^a^ | 0.69 (0.27–1.78) | 1.00 (Reference) | 4.32 (1.76–10.64) |
| AKI non-recovery |  |  |  |
| Participants, No. (%) | 9 (7.1) | 32 (9.0) | 8 (21.1) |
| Adjusted OR (95% CI) ^a^ | 0.77 (0.36–1.66) | 1.00 (Reference) | 2.69 (1.14–6.37) |

Abbreviations: AKI, acute kidney injury; CI, confidence interval; OR, odds ratio.

^a^ Adjusted for age, gender, baseline eGFR, hypertension, diabetes mellitus, EuroSCORE, NYHA class, contrast agents, ACEIs or ARBs, diuretics, statins, NSAIDs, cardiopulmonary bypass, type of surgery, surgery time, and intraoperative blood transfusion volume.

Table S6. Association between ΔScr group and secondary outcomes after excluding patients with serum creatinine ≥1.5 times baseline within 48 hours before surgery

| Secondary outcome | AKI status | Negative ΔScr (n=127) | Normal ΔScr (n=355) | Elevated ΔScr (n=38) |
| --- | --- | --- | --- | --- |
| In-hospital mortality |  |  |  |  |
| Adjusted OR (95% CI) a | With AKI | 4.75 (1.41‒16.02) | 1 (reference) | 1.36 (0.31‒5.95) |
|  | Without AKI | NA* | 1 (reference) | NA* |
| ICU LOS > 72 hours |  |  |  |  |
| Adjusted OR (95% CI) a | With AKI | 2.93 (1.25‒6.89) | 1 (reference) | 1.80 (0.76‒4.29) |
|  | Without AKI | 1.19 (0.65‒2.17) | 1 (reference) | 2.23 (0.54‒9.30) |

Abbreviations: AKI, acute kidney injury; CI, confidence interval; ICU LOS, intensive care unit length of stay; OR, odds ratio

a Adjusted for age, gender, baseline eGFR, hypertension, diabetes mellitus, EuroSCORE, NYHA class, contrast agents, ACEIs or ARBs, diuretics, statins, NSAIDs, cardiopulmonary bypass, type of surgery, surgery time, and intraoperative blood transfusion volume.

* The analysis could not be performed due to insufficient events.
